# Quorum Sensing Signal Synthesis May Represent a Selective Advantage Independent of Its Role in Regulation of Bioluminescence in *Vibrio fischeri*

**DOI:** 10.1371/journal.pone.0067443

**Published:** 2013-06-18

**Authors:** Grace Chong, Önder Kimyon, Mike Manefield

**Affiliations:** Centre for Marine BioInnovation (CMB), School of Biotechnology and Biomolecular Sciences (BABS), University of New South Wales (UNSW), Sydney, Australia; Tel Aviv University, Israel

## Abstract

The evolution of biological signalling systems and apparently altruistic or cooperative traits in diverse organisms has required selection against the subversive tendencies of self-interested biological entities. The bacterial signalling and response system known as quorum sensing or Acylated Homoserine Lactone (AHL) mediated gene expression is thought to have evolved through kin selection. In this *in vitro* study on the model quorum sensing bioluminescent marine symbiont *Vibrio fischeri*, competition and long-term sub culturing experiments suggest that selection for AHL synthesis (encoded by the AHL synthase gene *luxI*) is independent of the quorum sensing regulated phenotype (bioluminescence encoded by *luxCDABE*). Whilst results support the hypothesis that signal response (AHL binding and transcriptional activation encoded by the *luxR* gene) is maintained through indirect fitness benefits (kin selection), signal synthesis is maintained in the *V. fischeri* genome over evolutionary time through direct fitness benefits at the individual level from an unknown function.

## Introduction

The behaviour of biological systems requiring forms of signalling and cooperation between individual partner organisms have long garnered interest from evolutionary biologists [1]. The interest generally stems from the challenge of explaining how cooperative traits resist corruption by the self-interest of partner individuals. So-called ‘cheating’ has received much attention in the scientific literature in diverse organisms including microbes and several possible strategies for microbial cheater control have been postulated [2]–[4].

Bacterial quorum sensing is a term used to describe the release of signalling molecules by bacterial cells into the extracellular environment and a response to the accumulation of the signalling molecule through changes in transcription and hence phenotype [5]. The phenotypes regulated by quorum sensing are typically ecologically relevant only when expressed in concert by large populations of cells [6], [7]. In this way both the concerted production of signalling molecules and the orchestrated expression of its regulated phenotypes are distinct cooperative traits that have survived the subversive tendencies of self-interested individuals over evolutionary time.

*N*-acyl-L-homoserine lactone (AHL) mediated gene expression is one such bacterial quorum sensing system that has received an enormous amount of attention primarily owing to the direct role of many of the regulated phenotypes to human and plant infection [5]. AHL mediated gene expression involves the production of AHLs by an AHL synthase enzyme most commonly encoded by a homologue of the *luxI* gene of the marine symbiont *Vibrio fischeri*. AHLs typically diffuse through cell membranes and increase in concentration in the local extracellular environment if there are many producing cells in one location [8] and there are barriers to diffusion preventing AHL loss from that location [9]. Once a threshold intracellular concentration is reached, AHLs interact directly with transcriptional regulators encoded by homologues of the *luxR* gene of *V. fischeri* resulting in a transcriptional response. In the case of *V. fischeri*, in which AHL mediated gene expression was discovered, the transcriptional response encodes for the production of light that forms the basis of symbiotic interactions with marine fish and the bobtail squid *Euprymna scolopes* [10], [11].

In recent years, experiments have been conducted to investigate the vulnerability of the two distinct cooperative activities constituting quorum sensing: 1) the production of a communal stimulatory pool of AHLs and 2) the orchestrated expression of a communally beneficial phenotype [4]. Specifically, signal synthesis and response mutants were cultured *in vitro* in media requiring the communally beneficial phenotype (elastase production) for growth. The results suggested that exploitative individuals of the opportunistic human pathogen *Pseudomonas aeruginosa* can avoid both the cost of contributing to the communal AHL pool or the cost of contributing to the communally beneficial phenotype. It was concluded that kin selection is responsible for maintenance of genes encoding AHL production and response.

Additionally, there has been recent interest in the evolution of the *V. fischeri lux* system. Bose and colleagues have shown *in vitro* that bioluminescence represents a cost [12] and that *V. fischeri* strains isolated from different environments have diverse bioluminescence output [13]. The variations in the light output were attributed to rapid evolution in the intergenic sequences between the divergently transcribed *luxR* and *luxI* genes controlling their transcription rather than mutations in the quorum sensing genes themselves.

In related work, Schuster and colleagues [14] developed an exquisite experimental evolution model in which the luminescent output of free-living (strain WH1) or fish associated (strain MJ11) *V. fischeri* isolates decreased over approximately 300 generations in association with *E. scolopes*. Whilst no sequencing was conducted to link the changes in light output to genetic mutation, an AHL bioassay and responses to exogenous AHL addition revealed, for the most part, that changes were not derived from changes in AHL synthesis or response [14]. The evolution of the bioluminescent phenotype in *V. fischeri* in the absence of selection for it *in vitro* has not been investigated.

Differences in the specific hypotheses tested and approaches used in studies on the evolution of AHL mediated gene expression in *P. aeruginosa* and *V. fischeri* make it difficult to assess whether observations made are universal across AHL signalling systems or if there are fundamental differences driving their evolution. In this study a combination of *in vitro* approaches were used to investigate the stability of AHL mediated gene expression in *V. fischeri*, including competition and long-term subculturing experiments in the absence of selection for the regulated phenotype. Evidence is presented supporting the hypothesis that AHL synthesis is anchored in bacterial genomes through a fitness benefit to individual cells independent of group behaviour.

## Methods

### Batch culture growth of *V. fischeri* MJ1 wild type and MJ211 *luxI^–^* mutant in isolation and in co-culture

To compare growth of *V. fischeri* MJ1 [15] and the MJ1 derived *luxI* knockout mutant *V. fischeri* MJ211 [16] in isolation, batch cultures were established in triplicate from overnight precultures grown in 100 ml of LB20 medium (1% w/v tryptone, 0.5% w/v yeast extract and 2% NaCl w/v) in a 250 ml Erlenmeyer flask at 30°C and 150 rpm from an initial optical density of 0.01 at 600 nm. Experiments were conducted in the presence and absence of 5 µM *N*-3-oxohexanoyl-L-homoserine lactone in ethanol purchased from the laboratory of Paul Williams (Nottingham, UK). The unpaired t-test was performed on triplicate readings from all time points to identify statistically significant differences (p<0.05). Triplicate sets of optical density readings from wild type and mutant cultures for each time point were regarded as independent and assumed to be identically normally distributed.

**Figure 1:**
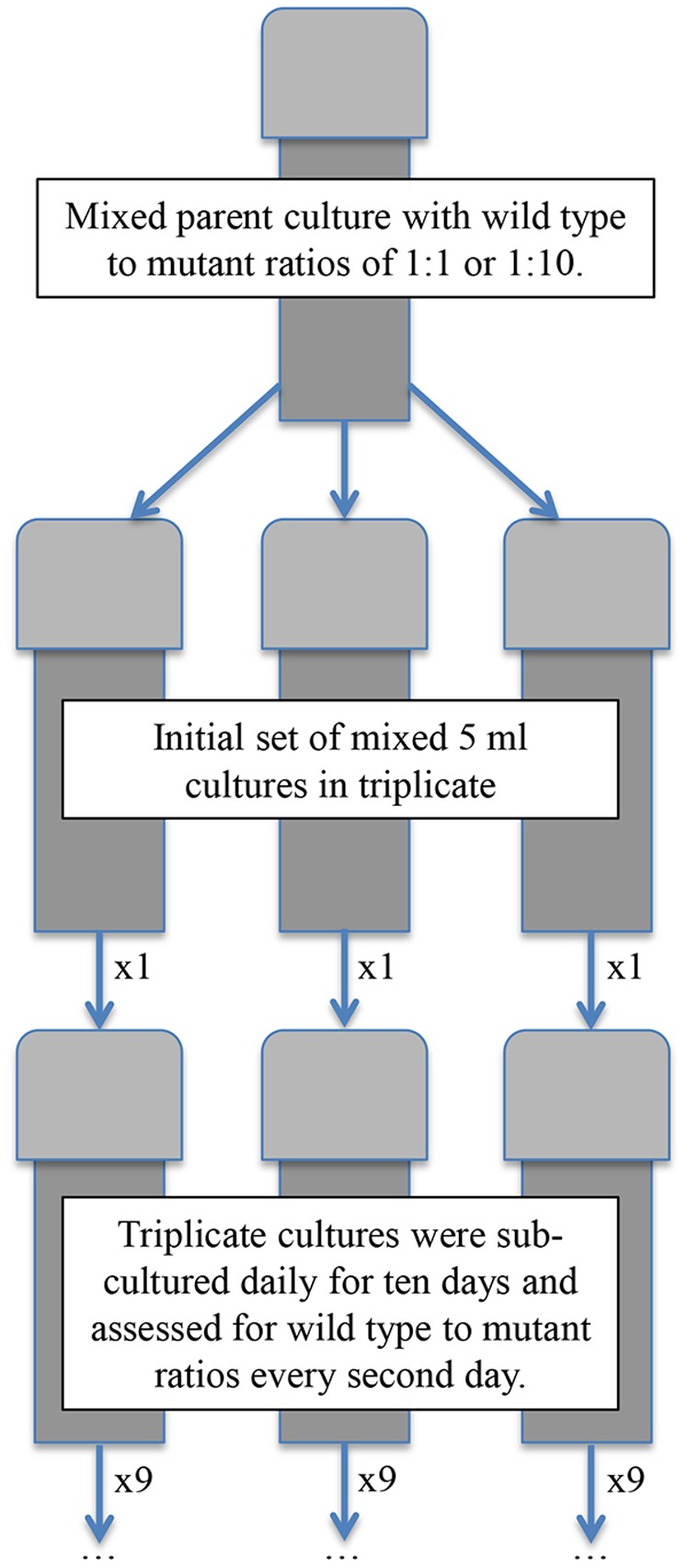
Experimental design for competition experiments. Mixtures of *Vibrio fischeri* wild type (MJ1) and *luxI* mutant (MJ211) strains at optical density based starting ratios of 1∶1 or 1∶10 were inoculated (100 µl) into 5 ml of fresh media in triplicate. Each replicate culture was subsequently subcultured daily for ten days and wild type to mutant ratios determined every second day.

Competition between the wild type and mutant strain was assessed in triplicate 5 ml aliquots in 20 ml plastic screw-capped vials in sequential batch co-culture with starting wild type to mutant ratios of 1∶1 or 1∶10 based on optical density with a starting density of 0.01. Every 24 h over a ten-day period 100 µl of stationary phase culture was transferred to 5 ml of fresh LB20 and the cell concentration of each competitor quantified through plating and counting of bioluminescent (MJ1) and non-bioluminescent (MJ211) colonies. The experimental design is illustrated in Figure 1.

### *In vitro* evolution of bioluminescence in *V. fischeri* MJ1

From a fresh LB20 agar plate inoculated from a glycerol freezer stock maintained in the Centre for Marine BioInnovation (University of NSW, Sydney, Australia) ten colonies of wild type *V. fischeri* strain MJ1 were used to inoculate 5 ml of AB_(VH)_ medium in 20 ml plastic screw-capped vials and cultured for 24 h shaking at 30°C and 150 rpm. An aliquot (100 µl) of each of the ten stationary phase cultures was then transferred to 5 ml of fresh AB_(VH)_ medium and incubated as above for another 24 h. This sub-culturing routine was carried out every 24 h for 325 days. AB_(VH)_ medium consisted of per liter: 0.3 M NaCl, 0.05 M MgSO_4_.7H_2_O, 2 g/L casamino acids (0.2%), 10 mM KH_2_PO_4_, 1 mM L-arginine, 2% v/v glycerol, 10 ng/ml riboflavin and 1 µg/ml thiamine [17]. Every 10 – 20 days ten 200 µl aliquots were taken from each of the ten cultures at the end of the logarithmic phase of growth (approximately 15 h into the batch culturing cycle, OD_600_  =  0.18) and bioluminescence was quantified using a microtiter plate luminometer (Wallac Victor2).

### Sequencing dark cultures

Dark *V. fischeri* cultures were plated out and at least five colonies were randomly picked for sequencing of *luxR*, *luxI*, *ainR*, *ainS* genes and the *luxR*-*luxI* intergenic region using primer sets listed in Table 1. Primers for genes were designed using the *V. fischeri* ES114 genome to cover the entire coding region. The PCR conditions used for the amplification of genes were an initial denaturation at 95°C for 10 min, followed by 25 cycles of 95°C for 15 s, 56°C for 30 s, and 68°C for 2 min, and a final extension cycle of 5 min at 68°C. PCR products were cleaned using the QIAquick® PCR Purification Kit (Qiagen Pty. Ltd., Australia) and quantified using a Nanodrop spectrophotometer.

**Table 1:** Primers used for sequencing *V. fischeri* bioluminescence regulatory genes.

Target	Primer	Sequence (5′- 3′)
*luxR*	Lux8F^a^	TTCCTGGTTCAGAGCCTCAT
	Lux1445R^a^	GCACTCTGTTGACCAAGCAA
*luxI*	Lux1220F^a^	GCAATTCCATCGGAGGAGTA
	Lux2019R^a^	CACTTTTCCATCGTTGACCA
*ainR*	Ain1064F^b^	TGGCTCTTCTTTGACGGTTT
	AinSR^b^	AGCTTAAAGAAATTAATGCTCGTCAG
*ainS*	Ain20F^b^	CACGACGAGAACCAAGACCT
	Ain1243R^b^	AACGAATTGCTTCGCATACC
*luxR*-*luxI*	EVS109^c^	CCGCCCTAGGTTATTCAGATAAGCATTGATTAATATC
intergenic region	EVS110^c^	CCGCCCTAGGGCATGCTTAACCTCTATACTCCTCCGATGGAA

a Primers and base numbering based on *luxR* and *luxI* (Genbank AF170104.1), ^b^Primers and numbering based on *ainR* and *ainS* (Genbank L37404), ^c^ [38].

A sequencing reaction was set up for one of the primers which contained at least 100 ng of PCR products, 1 µl BigDye Terminator 3.1 (Applied Biosystems, United Kingdom), 10 pmol of primer, 3.5 µl 5x buffer (Applied Biosystems, United Kingdom) and molecular grade H_2_O (Eppendorf, Australia) to a total of 20 µl. The conditions used for the sequencing reaction were an initial step at 96°C for 1 min, followed by 25 cycles of 96°C for 10 s, 50°C for 5 s and 60°C for 4 min. The sequencing reactions were cleaned up by ethanol precipitation and sequenced with an ABI Prism BigDye kit (Perkin-Elmer Applied Biosystems, Foster City, Calif.) and an

ABI model 310 genetic analyser (Perkin-Elmer Applied Biosystems) at the Ramaciotti Centre at the University of NSW, Sydney, Australia.

### *In vitro* evolution of bioluminescence in *V. fischeri* MJ1 with *luxR* harboured on a plasmid

To assess the evolution of the *V. fischeri* MJ1 bioluminescence phenotype in the presence of multiple copies of the *luxR* gene a multi-copy plasmid was constructed harbouring the functional *luxR* gene from *V. fischeri* MJ1 and transferred into a dark *V. fischeri* MJ1 lineage (Lineage 10). The wild-type *luxR* gene was amplified using pJBA132 (*p*ME6031-*luxR*-P*_luxI_*-RBSII-*gfp*(ASV)-T_0_-T_1_; Tc^r^; [18]) as the template and primers LuxR-SacI (5′-TTGCCGAGCTC TTTTGCCCAACAGAAAAAGC-3′) and LuxR-KpnI (TTTGCCGGTACC CTCCCTTGCGTTTATTCGAC) incorporating two restriction enzyme sites (underlined) flanking the 1.1 kb PCR product including the *luxR* promoter region. A 5 kb SacI-KpnI fragment from *p*LS6 (Cm^r^, *lacZ* expression vector derived from pSUP102; [19]) containing a chloramphenicol resistant gene was ligated to the SacI-KpnI digested amplified PCR fragment (1.1 kb) containing the *luxR* gene. The ligated construct (pLS6luxR) was then chemically transformed into competent *Escherichia coli* SM10 cells (*Thi, thr, leu, tonA, lacy, supE, resA*::RP4-2-Tc^r^::Mu, Km^r^; [20]), and used as the donor for conjugal transfer of the construct into recipient *V. fischeri* MJ1 Lineage 10 (*luxR* ^–^) cells via filter mating [21]. *V. fischeri* MJ1 Lineage 10 cells harbouring pLS6LuxR were selected on LB20 plates containing chloramphenicol (2 µg/ml). The presence of the plasmid was confirmed in bioluminescent *V. fischeri* colonies by plasmid extraction, restriction digestion and gel electrophoresis using standard protocols.

To assess the loss of the bioluminescent phenotype, *V. fischeri* MJ1 Lineage 10 (*p*LS6LuxR) colonies were subcultured in 5 ml AB_(VH)_ media every 24 h for 150 days and monitored for changes in bioluminescence as above. As an additional assessment for dark mutants (including *luxI* mutants) appropriate dilutions of culture were plated out on LB20 (Cm) agar plates every 14 days and screened for the presence of non-bioluminescent colonies.

## Results

### Growth curve comparisons between *Vibrio fischeri* strain MJ1 and the AHL synthase mutant strain MJ211

It is generally believed that *N*-acyl-L-homoserine lactones (AHLs) are secondary metabolites that by definition play no role in cell-structure syntheses and energy transduction in the producer and are therefore not essential for growth and reproductive metabolism [22]. We began our investigation seeking to confirm that deletion of the AHL synthase gene *luxI* did not compromise the ability of *V. fischeri* to reproduce by comparing growth of the wild type (strain MJ1) and its AHL synthase mutant (strain MJ211) in batch aerobic cultures in the presence and absence of the cognate AHL *N*-3-oxohexanoyl-L-homoserine lactone (OHHL) at 5 µM.

**Figure 2:**
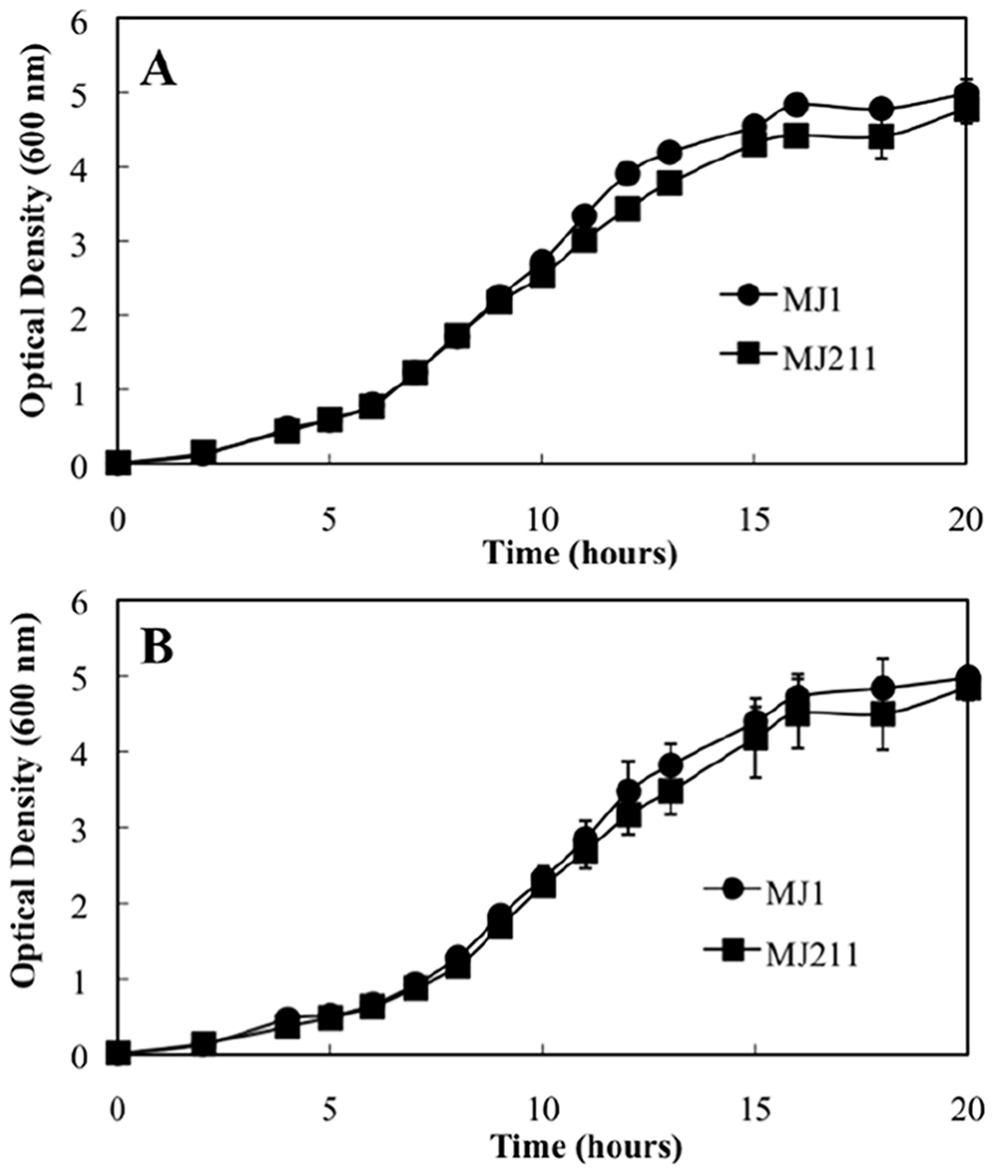
Impact of *luxI* deletion on growth of *Vibrio fischeri* in isolation. Batch culture growth of *V. fischeri* wild type strain MJ1 (circles) and *luxI* deficient mutant strain MJ211 (squares) in the absence (A) and presence (B) of 5 µM *N*-3-oxohexanoyl-L-homoserine lactone. Values presented are averages of triplicate cultures. Error bars represent standard deviation. Small statistically significant differences were late in the logarithmic phase of growth.

Figure 2 illustrates the average increase in optical density of triplicate batch cultures over time. This data reveals that the AHL synthase mutant has very similar growth characteristics to the wild type in batch culture. Small differences were however observed, with the AHL synthase mutant strain generating slightly lower optical densities than the wild type (Fig. 2A). Between 10 and 16 hours in the absence of exogenously added OHHL these differences were statistically significant (Unpaired T test, p <0.05). Statistically significant differences were also observed in the presence of OHHL, however these were observed earlier in the incubation at 4 and 8 hours (Unpaired T test, p <0.05; Fig. 2B). In conclusion, the *luxI* gene is clearly not essential for the growth of *V. fischeri* under these conditions but subtle differences in optical density were noted suggesting the *luxI* gene could confer a detectable fitness advantage.

### Growth competition between *Vibrio fischeri* strain MJ1 and the AHL synthase mutant strain MJ211

To further investigate the subtle differences observed in the growth characteristics of wild type *V. fischeri* and the AHL synthase mutant strain, both were subject to direct competition for resources by co-inoculation into triplicate aerobic batch cultures that were subcultured daily for 10 days. Each strain was quantified every second day by plate counting with the mutant strain distinguished from the wild type based on the luminescence of colonies. Note that the mutant *V. fischeri* strain MJ211 in co-culture will be exposed to the same extracellular concentration of AHLs as the AHL producing wild type strain MJ1. Hence it is assumed that in the same vessel both strains transcribe the *luxCDABEG* operon encoding luminescence to the same degree with comparable associated impacts on fitness.

**Figure 3:**
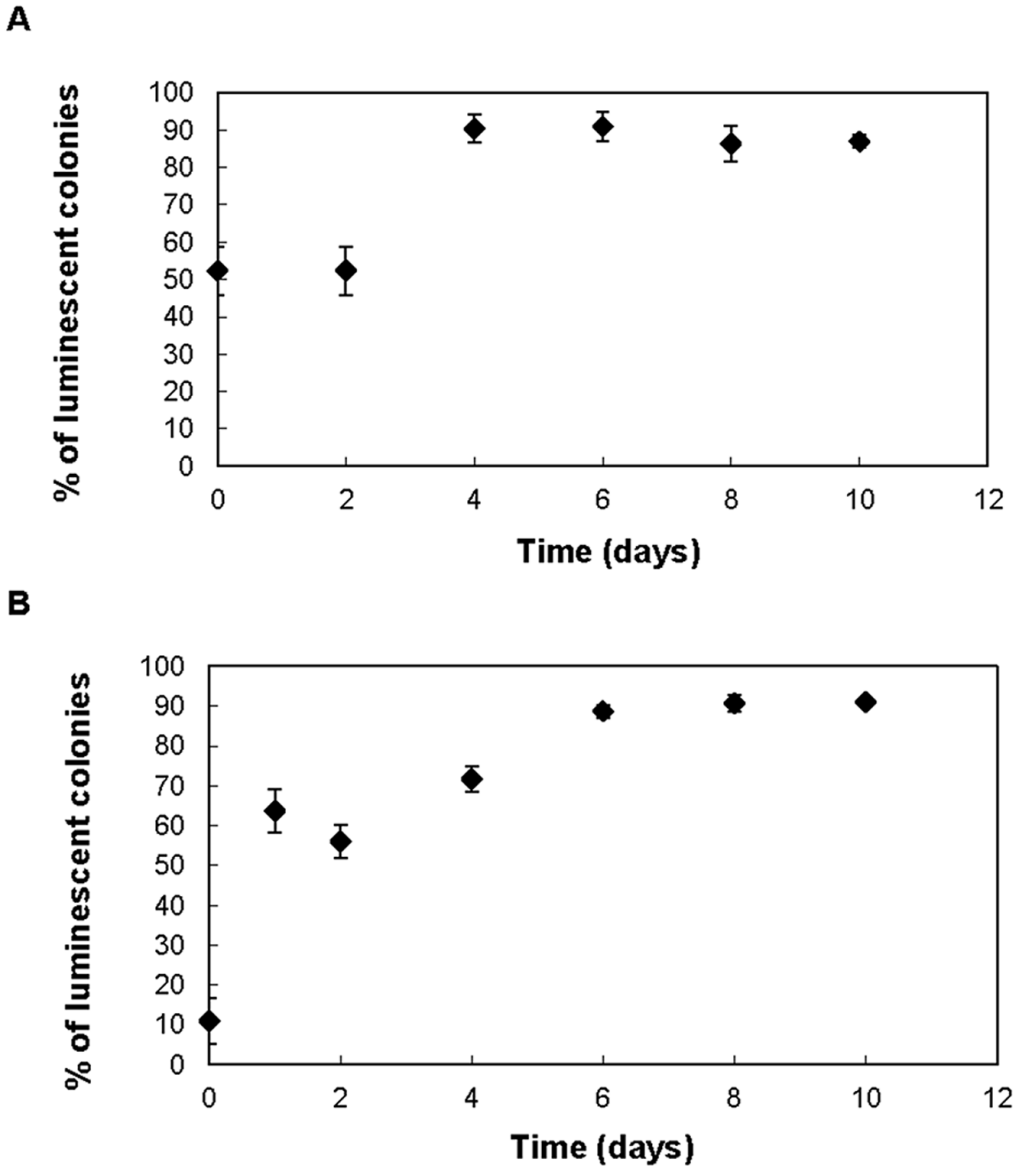
Impact of *luxI* deletion on competition in *Vibrio fischeri*. Percentage of luminescence colonies plated from mixed *V. fischeri* MJ1 (wild type) and MJ211 (*luxI* mutant) cultures subcultured daily for ten days. The starting ratio of MJ1 to MJ211 was either 1∶1 (Panel A) or 1∶9 (Panel B). Regardless of the initial ratio of wild type (MJ1) to mutant (MJ211) cells, the wild type lineage dominated the cultures within days suggesting the *luxI* gene represents a selective advantage independent of the bioluminescence phenotype. Average values from triplicate cultures are presented. Error bars represent standard deviation.

Figure 3 reveals that the luminescent wild type strain grew to dominate all cultures during the course of the experiment irrespective of the starting population ratios. From a starting ratio of 1∶1 it took four days for the ratio to reach 9∶1 (wild type:mutant). From a starting ratio of 1∶10 it took six days for the ratio to reach 9:1 (wild type:mutant). It is recognised that scoring dark colonies as a means of quantifying the *luxI* mutant MJ211 may include spontaneous mutants of the wild type in genes encoding bioluminescence (eg. *luxR*, *luxICDABEG*), although the likelihood is low given the short sub-culturing period. In effect this would overestimate the MJ211 counts and suggest the MJ211 population decline is even more rapid.

In conclusion, in culturing conditions under which there is no known selection for the AHL regulated bioluminescence phenotype, the wild type *V. fischeri* strain has a clear fitness advantage over the mutant. This result is in conflict with the suggestion that AHL production *per se* represents a net metabolic burden to producing cells. Further, this result suggests the activity of the *luxI* gene product confers a fitness advantage independent of the AHL regulated phenotype.

### Long term evolution of the quorum sensing system in *V. fischeri* MJ1

**Figure 4:**
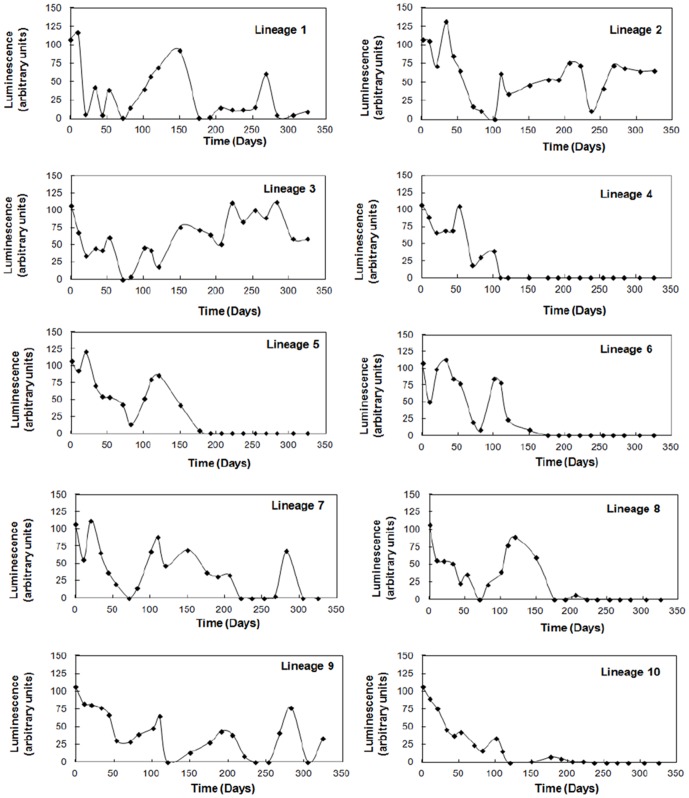
Fate of the bioluminescence phenotype during long term culturing in *Vibrio fischeri*. Relative bioluminescence output from ten *V. fischeri* MJ1 cultures, subcultured daily over 325 days. Large fluctuations are observed in bioluminescence in all cultures. Half of the cultures (4, 5, 6, 8 and 10) irreversibly lost the bioluminescence phenotype during the course of the experiment. Quorum sensing regulatory genes (*ainS*, *ainR*, *luxI* and *luxR*) and the *luxI*-*luxR* intergenic region were sequenced to investigate the cause of the loss of phenotype.

To explore the fate of the quorum sensing system in *V. fischeri* over thousands of generations, strain MJ1 was inoculated into ten identical aerobic batch cultures that were subsequently subcultured daily for 325 days (approximately 4000–5000 generations). In this experiment the cost of signal synthesis and response could be compared without interference from gene maintenance derived from selection for the regulated phenotype. The bioluminescent output of each culture was monitored every 10–20 days in the late-logarithmic phase of growth throughout the course of the experiment. Figure 4 illustrates the luminescence of each culture in relative light units and reveals fluctuations over several orders of magnitude in light output over time with striking differences observed between lineages. All cultures displayed decreases and increases in luminescence output, whilst five lineages (Lineages 4, 5, 6, 8 and 10) permanently lost the luminescent phenotype during the course of the experiment.

Observations on individual colonies derived from the cultures throughout the experiment revealed diversity in luminescence output (bright, dim and dark) in cultures showing variable luminescence over time. In contrast, colonies derived from lineages 4, 5, 6, 8 and 10 after they had permanently lost the luminescence phenotype were all dark suggesting dark mutants had taken over due to major fitness benefits. This expected result confimed that under the prevailing culture conditions the luminescent phenotype confers a net disadvantage to *V. fischeri* cells.

To assess whether the loss of luminescence was the result of mutations in genes encoding AHL synthesis or response, the *luxR*, *luxI*, *ainS* and *ainR* genes were sequenced along with the *luxI*-*luxR* intergenic region from at least five colonies in the 250–300 day period for each of the ten lineages. It was observed that four out of the five permanently dark lineages had base pair substitutions or deletions in the *luxR* gene. Lineage 5 had no mutations in any of the regions sequenced.

Lineage 4 had a four base pair deletion (ATTG) at the end of the sequence (bp 727–730 from the start of the coding sequence or 239–242 in Genbank AF170104.1) removing residue 243 (isoleucine) and creating a frameshift in the remaining 23 bp of the coding region. In a site directed mutagenesis study, Trott and Stevens [23] revealed that residue 243 was crucial for LuxR binding to DNA and activation of the *lux* operon, thus explaining the origin of the dark phenotype in this lineage.

Lineage 6 had a base pair substitution (GGA > AGA at bp 400 from the start of the coding sequence or bp 569 in Genbank AF170104.1) predicted to result in a change in the protein sequence at residue 134 from glycine (aliphatic) to arginine (basic). This residue forms part of the five-stranded anti-parallel ß5 sheet of the AHL binding cavity [24]. Whilst it is not a conserved residue amongst LuxR homologues, it sits in a cluster of physiologically conserved amino acids [24]. It is therefore likely that this mutation from an aliphatic to basic residue has resulted in loss of AHL binding activity.

Lineage 8 had a base pair substitution (TGC > TAC at bp 584 from the start of the coding sequence or bp 385 in Genbank AF170104.1) predicted to result in a change in the protein sequence at residue 195 from cysteine (sulfur containing) to tyrosine (aromatic). Residue 195 sits in the helix-turn-helix region of LuxR known for DNA binding [24]. This mutation therefore likely impacts on *lux* gene transcription by interfering with LuxR binding to the *lux* box.

Lineage 10 had undergone a base pair substitution (ATT > AGT at bp 728 from the start of the coding sequence or bp 241 in Genbank AF170104.1) predicted to result in a change in the protein sequence at residue 243 from isoleucine (aliphatic) to serine (hydroxyl containing). This is the same residue lost in Lineage 4 previously shown to be crucial for LuxR activity [23].

No mutations were observed for the *luxI*, *ainS* and *ainR* genes in all lineages and in contrast to the observations of Bose and colleagues [13] no mutations were observed in the intergenic region encoding the promoter regions for *luxI* and *luxR*. This result suggests there was selection against the action of the *luxR* gene product under the growth conditions tested. The same cannot be said of the *luxI* gene, adding further weight to the suggestion that expression of the *luxI* gene and activity of its product represents an unknown selective advantage to the cell.

### Evolution of bioluminescence with *luxR* carried on a multiple copy number plasmid

**Figure 5:**
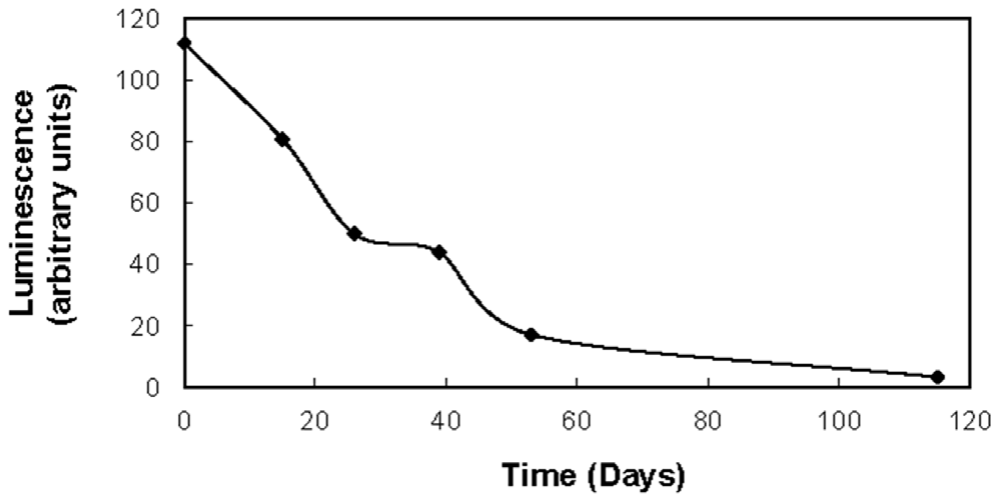
Impact of *luxR* complementation on bioluminescene during long term culturing of *Vibrio fischeri*. Relative bioluminescence output from the dark *V. fischeri* MJ1 Lineage 10 with a *luxR* mutation complimented with a functional *luxR* gene under control of its own promoter on pLS6luxR. Complementation with *luxR* initially restored the bioluminescence phenotype to wild type levels, however bioluminescence was observed to decrease during subsequent subculturing for 120 days.

It was reasoned that the absence of *luxI* mutants in the ten *V. fischeri* lineages may have resulted from competition from *luxR* mutants. Cells not responding to the signal would be expected to have a greater selective advantage than cells not producing the signal but still encoding a functional response. To address this, a plasmid (*p*LS6LuxR) with a functional *luxR* gene was inserted into a single clone from the permanently dark *V. fischeri* lineage 10 (*luxR* mutant) and subcultured daily for 150 days. The culture was monitored for luminescence over time. Figure 5 reveals that the light output decreased gradually over time. Individual cells were assessed for loss of *luxI* activity resulting in a completely dark phenotype by plating cells and scoring luminescence of the resulting colonies. No dark mutants were observed during the course of the experiment indicating the absence of *luxI* mutants. The cause of the gradual decline in the luminescence output of the culture is unknown. The fact that all colonies on plates displayed some level of luminescence supports the assertion that the loss of *luxR* is the primary mechanism by which *V. fischeri* cells opt out of expression of the cooperative bioluminescence trait.

## Discussion

Over the past 15 years significant attention has been given to the individual conflicts underlying the cooperative behaviours observed in AHL mediated gene expression in bacteria. This includes both the generation of a communal AHL pool requisite for expression of a trait for which there are ecological benefits only if it is performed *en mass* and the communal expression of that trait. In this short history, bacterial quorum sensing has provided insight into the evolution of signalling and other cooperative biological phenomena.

Initially, Brookfield [25] and Brown and Johnstone [26] developed bacterial population genetics models of quorum sensing evolution revealing parameter space in which the cooperative traits of signal production and orchestrated phenotype expression were stable despite the presence of signal production or response deficient mutants. It was reasoned that kin selection or indirect fitness benefits were responsible, given the high level of relatedness between clones in a bacterial population. In support of the assumption of existence of signalling deficient mutants in these theoretical works, empirical studies have been published describing the isolation of *P. aeruginosa* strains with mutations in their quorum sensing machinery, predominantly in *lasR* [27], [28]. Additionally, studies have been published exploring the fitness costs of signal production and functional gene expression and demonstrating conditions under which the proliferation of quorum sensing cheats is fostered [3], [4], [29]. In this study we have shown that in the absence of selection for the regulated phenotype, wild type *V. fischeri* cells have a higher reproductive fitness than cells lacking the *luxI* gene and that *luxI* mutants do not proliferate over thousands of generations in the presence or absence of functional *luxR* genes, while *luxR* mutants in contrast commonly arise. This suggests that the activity of the *luxI* gene represents a direct fitness benefit selected for independently of the bioluminescent phenotype in this quorum sensing model organism.

Diggle and colleagues [4] rightly pointed out that multiple functions of signalling molecules could alter the relative cost of AHL production, thereby providing an alternative explanation to kin selection for the maintenance of AHL production over evolutionary time. Interestingly, the three examples given to support the point (antibiotic, siderophore and immune modulatory activity) are all cooperative traits conferring indirect fitness benefits, requiring a dense population of cells for the selective advantage of the phenotype to be manifested. In all the literature relating quorum sensing to group selection no mention has been made of the possibility that AHL synthase activity represents a direct fitness benefit, *ie*. a selective advantage to individual *V. fischeri* cells independent of the indirect fitness benefits derived from cooperative group behaviour (bioluminescence phenotype).

The experiments conducted in this study do not shed light on what the selective advantage of AHL synthesis to individual *V. fischeri* cells could be although it is clear that it does not relate to activation of gene transcription through interaction with LuxR. An emerging hypothesis explaining the advantage of AHL synthesis stems from observed links between AHL regulated phenotypes and maintenance of redox homeostasis in the bacterial cytoplasm of *V. fischeri* and *Pseudomonas aeruginosa* [30]–[33].

The *luxI* gene encodes a 193 amino acid protein responsible for conversion of S-adenosylmethionine and hexanoyl-acyl carrier protein to *N*-3-oxohexanoyl-L-homoserine lactone, 5′-adenosylmethionine and apo-Acyl carrier protein [34]. Because *N*-3-oxohexanoyl- L-homoserine lactone can diffuse through cell membranes, its synthesis represents a path for excess reducing equivalents to leave the cytoplasm.

Traditional batch culturing conditions using rich media formulations, including those used in this study, are a good example where reduced organic carbon is supplied in excess and oxygen (electron sink) rapidly becomes limiting as cell densities increase. Biofilms are another example in which cells are exposed to excess reducing power (polysaccharides, proteins, glycoproteins, glycolipids and extracellular DNA) in extracellular polymeric matrices [35] with constricted access to electron acceptors based on consumption and diffusion limitations [36], [37]. Future experiments will address the hypothesis that AHL production and release represents a means of removing excess reducing power from the cytoplasm.

In summary, this study has shown that in the absence of selection for the bioluminescence phenotype *V. fischeri* MJ1 wild type cells outcompete *V. fischeri* MJ211 *luxI* deficient cells and that *luxI* mutations do not arise during subculturing over thousands of generations in the presence or absence of functional LuxR. The appearance of *luxR* mutants in our experiments is congruent with the hypothesis that this gene is maintained in nature through kin selection. In contrast, these results lend credence to the suggestion that *luxI* is maintained in the *V. fischeri* genome, not by kin selection, but by an unknown function of relevance to individual cells.
